# Updates in the Management of Coronary Artery Disease: A Review Article

**DOI:** 10.7759/cureus.50644

**Published:** 2023-12-16

**Authors:** Archit Bansal, Kishore Hiwale

**Affiliations:** 1 Pathology, Jawaharlal Nehru Medical College, Datta Meghe Institute of Higher Education and Research, Wardha, IND

**Keywords:** coronary artery disease, therapies, treatment, advancements, management

## Abstract

Coronary artery disease (CAD) remains a significant health challenge, imposing substantial burdens on individuals and healthcare systems worldwide. CAD's impact stems from artery narrowing and blockage, leading to severe complications like heart attacks and heart failure. Collaborative efforts by researchers, professionals, and governments have fostered advancements in comprehending and managing this cardiovascular ailment. Evolving CAD management embraces modern diagnostics, cutting-edge pharmaceuticals, invasive procedures, lifestyle modifications, and cardiac rehabilitation. This comprehensive approach aims to amplify outcomes and elevate the quality of life for CAD-affected individuals. This review delves into innovative treatments, pivotal breakthroughs, and recent trends in clinical practices that collectively shape CAD management. The exploration encompasses novel diagnostic technologies enabling early detection and risk assessment. Moreover, it investigates recent breakthroughs in medications that profoundly impact platelet disorders, lipid reduction, and angina. Precision medicine's role in tailoring treatment strategies based on patient characteristics is thoroughly examined. Advances in invasive procedures, like percutaneous coronary intervention (PCI) and coronary artery bypass grafting (CABG), have revolutionised coronary revascularisation, substantially improved long-term outcomes, and reduced restenosis rates. The increasing significance of lifestyle changes and cardiac rehabilitation in CAD management, augmenting treatment options and patient recovery, are meticulously scrutinized. While these strides are pivotal, research continues to chart new paths in CAD management, from innovative drugs to collaborative multidisciplinary care models. Staying attuned to the latest advancements and embracing a patient-centric approach can collectively reduce CAD's impact and facilitate the lives of those grappling with this chronic cardiovascular disorder.

## Introduction and background

**Figure 1:**
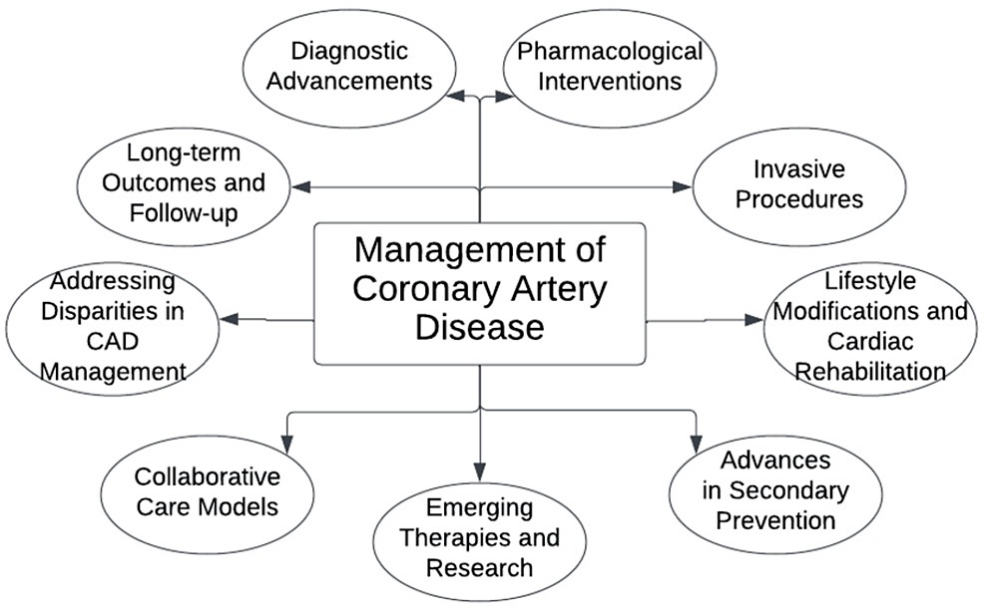
Subtopics under the management of coronary artery disease Image Credit: Author

Coronary artery disease (CAD) continues to pose a health challenge, impacting numerous individuals and placing strain on healthcare systems worldwide. The narrowing and blockage of arteries due to CAD can lead to complications like heart attacks and heart failure. Over time, dedicated efforts from researchers, professionals, and governments have contributed to advancements in understanding and managing this cardiovascular condition [1]. The management of CAD has changed, adopting an approach that incorporates modern diagnostic technologies, cutting-edge pharmaceutical medications, invasive procedures, advances in prevention, emerging therapies, collaborative models, long-term outcomes with follow-ups, and increased emphasis on lifestyle modifications and cardiac rehabilitation (Figure 1). This comprehensive approach aims to enhance CAD patients' outcomes and overall quality of life. This review will explore the treatment innovations for artery disease while delving into recent breakthroughs that have influenced clinical practices and improved patient care [2].

Additionally, we will examine advancements in techniques that provide healthcare providers with tools for early detection and risk assessment. Moreover, we explore the range of treatments available today, including innovative medications that have significantly transformed the management of platelet disorders, lipid reduction, and angina. Our discussion primarily focuses on comprehending the role played by precision medicine in tailoring treatment approaches based on patient characteristics [3]. The advancements in procedures, like percutaneous coronary intervention (PCI) and coronary artery bypass grafting (CABG), have revolutionised coronary revascularisation and resulted in better long-term outcomes and decreased restenosis rates. Furthermore, we analyze the growing importance of lifestyle changes and cardiac rehab centres in treating CAD, showing their ability to supplement treatment options and improve patient recovery and well-being. While these developments are significant, ongoing research opens new vistas in CAD management, from new medicines to multidisciplinary collaborative care models promoting teamwork [4]. We can work together to reduce the impact of CAD and improve the future for those affected by this chronic cardiovascular disease by staying updated on the latest advancements and using a patient-centred approach.

## Review

Methodology

For gathering all the required papers for this review article, a thorough literature search technique was needed in the methodology section of this narrative review. Google Scholar and PubMed were the two electronic databases accessed. The following keywords were used: "therapies," "treatment," "advancements," " management," and "coronary artery disease." All the authors unanimously carried out a thorough search.

**Figure 2:**
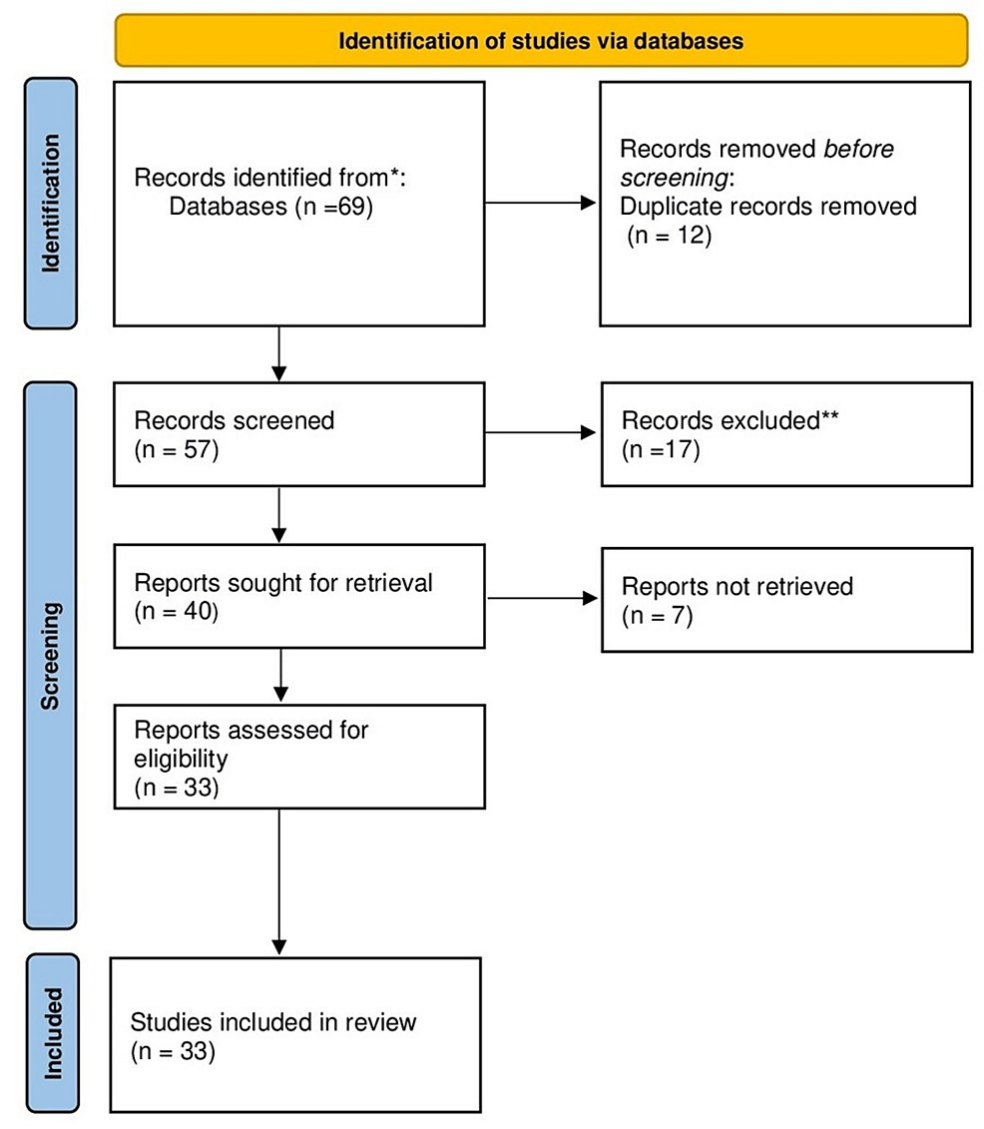
PRISMA methodology PRISMA: Preferred Reporting Items for Systematic Reviews and Meta-Analyses

The inclusion of both original research articles and review papers was taken into consideration. To ensure that the inclusion of studies satisfies the established criteria, the selection procedure comprised of evaluating article titles, abstracts, and full texts. Conflicts over the choice of studies were settled by author consensus. The method for choosing the articles we utilized in our study is shown in Figure 2.

Diagnostic advancements

CAD continues to be a leading cause of morbidity and death worldwide, with such proliferation calling for the ongoing advancement of analysis techniques to enhance early discovery and threat stratification. These testimonials highlight current breakthroughs in the management of CAD, with a concentration on non-invasive imaging methods, biomarkers, and customised threat accounts.

Non-invasive imaging methods have considerably advanced the medical diagnosis of coronary artery conditions and have enabled the exact visualisation of the coronary composition and myocardial features [5]. Specifically, coronary computed tomography angiography (CCTA) has become an effective device for the non-invasive examination of coronary arteries because it provides high-resolution photos and enables the precise discovery of coronary constriction and plaque. Cardiac magnetic resonance (CMR) imaging is likewise valued for its ability to evaluate myocardial feasibility perfusion and other features that assist in danger stratification and therapy preparation.

Biomarkers have emerged as promising tools for early CAD diagnosis and risk assessment. Their presence serves as a guarantee in very early CAD medical diagnoses and danger analyses. Highly sensitive troponins, for instance, have transformed the detection of myocardial damage by facilitating the early identification of patients with acute coronary syndromes. Additionally, researchers have explored new inflammatory markers, such as C-reactive protein and interleukins, to predict CAD progression and adverse cardiovascular events [6].

Personalised risk profiling, which encompasses genetic testing and advanced risk assessment tools, has ushered in an era of precision medicine in CAD management. Uncovering the genetic variants associated with susceptibility and response to CAD allows for tailored treatment strategies that cater to individual patient needs. Moreover, risk prediction models that integrate clinical, genetic, and lifestyle factors have been found to assist in identifying individuals at high risk of CAD, thereby enabling timely intervention and preventive measures. 

Despite these remarkable advances, challenges still exist in promoting the widespread adoption of these diagnostic strategies. Access to advanced imaging techniques, associated costs, and required operating expertise must be addressed. Further studies assessing these diagnostic tools' clinical utility and cost-effectiveness in routine CAD management are warranted [7].

Pharmacological interventions

In the last few years, brand-new antiplatelet strategies have reinvented how CAD is addressed and treated. Dual antiplatelet treatment (DAPT) with painkillers and P2Y12 receptor agents, such as clopidogrel, prasugrel, and ticagrelor, have become key approaches to caring for individuals with PCI [8]. These recently discovered mechanisms, specifically ticagrelor, have demonstrated greater effectiveness in treating major adverse cardiovascular events (MACE) and stent apoplexy, providing substantial benefits over traditional treatments. Breakthroughs in lipid-lowering treatments have also revealed considerable success. The development of proprotein convertase subtilisin/kexin kind 9 (PCSK9), an innovative medication course, has offered a reliable method for dealing with hypercholesterolemia, specifically in domestic cases or statin intolerance. When contributing to statin treatment, PCSK9 treatments have been associated with substantial decreases in low-density lipoprotein (LDL) cholesterol and cardiac events [9].

There have also been advancements in angina medications, with the development of newer agents, such as ranolazine, offering alternative treatment options. Due to its unique mechanism of action, which involves the inhibition of late sodium currents, ranolazine has shown efficacy in reducing tension-related chest pain and improving exercise tolerance, making it a valuable addition to traditional angina therapies. In addition, precision medicine has gained momentum in managing CAD, enabling individualised drug therapy based on genetic testing and risk profiling. Tailoring pharmacological interventions to a patient's genetic makeup and associated risk factors can improve treatment efficacy and minimise side effects, and the success of such approaches has launched a new era in personalised CAD treatment [10].

Invasive procedures

PCIs have seen substantial development in recent years. The introduction of next-generation drug-eluting stents has transformed worldwide strategies for coronary revascularisation. These stents are coated with medications that prevent restenosis, which significantly reduces the incidence of stent restenosis and the need for repeat interventions [11]. Additionally, bioresorbable scaffolds have emerged as a preferred option to typical steel stents, as they progressively liquify with time and are thus likely to bring back vascular features. Intravascular imaging strategies have become essential to PCI treatments. Intravascular ultrasound (IVUS) and optical comprehensibility tomography (OCT) offer high-resolution, real-time imaging of coronary arteries, thereby helping with more precise strengths, opportunities, aspirations, and result (SOAR) analysis and ideal stent positioning. The ability to image plaque attributes and stent growth has enhanced treatment success and led to long-lasting results. Minimally intrusive methods have likewise received more attention in CAD monitoring. A transradial approach, in which the procedure is performed through the carpal artery, has led to fewer bleeding complications and better patient comfort than the traditional femoral approach [12].

On top of these developments, robot-assisted PCI has demonstrated the capacity to boost driver accuracy and minimise exposure to direct radiation during treatment. Although PCI remains the mainstay of CAD treatment, CABG can be an essential option for select patients. Advances in surgical techniques, such as off-pump CABG and total arterial revascularisation, have reduced perioperative complications and improved graft capacity [13].

Lifestyle modifications and cardiac rehabilitation

A comprehensive modification of one's lifestyle is critical to avoid further complications due to CAD. Current research has highlighted the value of a heart-healthy diet regimen, routine workouts, smoking cessation, and general decreases in tension [14]. Integrating these changes with the use of drug therapy can minimise certain cardiac risks, such as high blood pressure, dyslipidaemia, and weight problems. Heart rehabilitation programmes have made considerable improvements in providing customised and structured treatments for individuals with CAD. These programmes include exercise, education, counselling to improve physical fitness, adherence to prescribed protocols, and psychosocial support. Technological developments have made remote monitoring and rehabilitation even more straightforward, allowing rehabilitation services to be available, even in remote or underserved areas [15].

Generally, studies have highlighted the long-term benefits of cardiac rehabilitation in reducing recurrent cardiovascular events and all-cause mortality. Individualised and patient-centred approaches have improved patient compliance with cardiac rehabilitation programmes, ensuring the protocol is tailored to individual needs and preferences. However, there are still challenges in adopting significant lifestyle changes and fully engaging in cardiac rehabilitation. Limited access to rehabilitation programmes, especially in low-income areas, and a lack of patient commitment to long-term lifestyle changes are ongoing challenges [16]. Addressing these challenges requires collaboration between healthcare providers, policymakers, and communities to create more affordable and accessible rehabilitation services.

Advances in secondary prevention

A crucial aspect of the secondary prevention of CAD involves effectively managing the modifiable risk factors that influence the progression and recurrence of the condition. In this context, evidence-based guidelines and treatment protocols have been established to provide healthcare professionals with more precise targets and therapeutic options [17]. Pharmaceutical developments have led to the introduction of novel lipid-lowering treatments, such as PCSK9 approaches and mixed treatments, which have revealed considerable efficiency in accomplishing reduced LDL cholesterol levels alongside conventional statin treatment. New antiplatelet agents and antithrombotic drugs have been developed to improve secondary prevention strategies in patients with a history of acute coronary syndrome. In addition to pharmacological measures, lifestyle changes play a crucial role in secondary prevention. Innovative digital health solutions and telemedicine have been helpful in this respect by facilitating remote monitoring and individualised interventions, allowing patients to adopt healthier habits and follow treatment plans more effectively [18]. Additionally, cardiac rehabilitation programmes have evolved to incorporate comprehensive lifestyle interventions, including exercise, nutritional counselling, and stress management. They have shown significant benefits in improving patient outcomes and reducing future cardiovascular events. Another significant advancement in secondary prevention is the emphasis on individualised risk assessment and treatment plans. Genetic testing and biomarker assessments have allowed doctors to identify high-risk patients and tailor their treatments accordingly. This approach optimises pharmacotherapy, invasive procedures, and lifestyle interventions based on a patient's genetic susceptibility and underlying pathophysiology [19].

Emerging therapies and research

Genetic treatments and gene-modifying strategies have become prospective game-changers in CAD therapy. These treatments target certain hereditary aspects that add to the advancement of CAD, ensuring customised and accurate treatments. Early studies have shown encouraging results, indicating the potential to reverse the progression of the disease and reduce the burden of atherosclerotic plaques [20]. Although these approaches are still in their infancy and require further investigation, they offer hope for people with a genetic predisposition to CAD and treatable cases that have not responded adequately to conventional therapies. Targeted therapies tailored to specific CAD subtypes are another exciting research avenue, wherein treatments addressing the unique pathophysiological mechanisms underlying the different subtypes are developed, recognising the heterogeneity of CAD presentations. This approach can improve treatment efficacy and reduce side effects by better tailoring interventions to each patient's disease characteristics. Regenerative medicine and stem cell therapy are gaining attention as potential ways to repair and regenerate damaged heart tissue in patients with CAD [21]. Preclinical studies and early clinical trials have demonstrated promising results in improving heart muscle function and reducing scar tissue formation. As these therapies progress in more extensive trials, they may revolutionise the management of CAD by providing restorative and regenerative options for patients with extensive CAD. Among these promising advances, however, are challenges. New therapies' long-term safety, cost-effectiveness, and scalability require thorough evaluation before clinical introduction. In addition, ensuring equal access to these innovative treatments for all patients is essential to avoid widening health inequalities [22].

Collaborative care models

Collaborative care models incorporate a selection of medical care experts consisting of cardiologists, medical care doctors, registered nurses, pharmacologists, nutritional experts, and rehab professionals who collaborate as a natural group to attend to the varied requirements of people with CAD. These models promise smooth interactions, shared decision-making, and collaborative activity, ensuring people get thorough and personalised treatment. One significant benefit of joint treatment designs is the capability to apply a multidisciplinary method to monitoring CAD [23]. Each team member brings specialised knowledge that enables a comprehensive assessment of the patient's condition. For example, cardiologists can focus on the medical management of CAD, nutritionists can help patients implement heart-related eating habits, and rehabilitation specialists can create customised exercise plans. This comprehensive method enhances therapy results and assists in addressing threats and way-of-life elements.

Furthermore, collaborative models enhance a person's education, learning, and self-care. Through regular interaction with different medical care specialists, clients get a much deeper understanding of their problem, therapy alternatives, and the way-of-life modifications required to deal with CAD effectively [24]. With knowledge and support, patients are more likely to adhere to prescribed medications, lifestyle changes, and follow-ups, leading to better long-term compliance and overall prognosis. In addition, collaborative care models promote early detection and timely intervention. Regular communication between team members allows for the quick identification of potential complications or changes in the patient's condition, which allows for quick changes in treatment plans. This proactive approach can prevent the progression of the disease and reduce the risk of side effects. However, successfully implementing collaborative care models requires effective communication, a streamlined workflow, and a commitment to professional collaboration. Barriers such as time constraints, resource allocation, and organisational culture can be complex but can be overcome with strong leadership, training, and support [25].

Addressing disparities in CAD management

Initiatives to eliminate disparities in CAD management have progressed over the last few years. Raised understanding projects, as well as targeted education and learning programmes, have aided in boosting public awareness about CAD threat variables and preventive measures. Furthermore, efforts to increase accessibility to healthcare and cardio testing in underserved locations have contributed to increased detection and prompt treatment. However, there are still significant gaps in resolving disparities in CAD management [26]. Socioeconomic factors critically determine access to quality healthcare, and marginalised communities often face barriers to seeking appropriate medical care. Limited access to preventive measures such as healthy food and recreational activities may exacerbate coronary heart disease (CHD) risks in vulnerable populations. Cultural competence and sensitivity in CAD management are important aspects that require more attention. Healthcare providers must have the necessary skills to understand and respond to the unique needs of different patient populations [27]. Adapting management strategies by considering cultural beliefs and practices can improve patient engagement and adherence. In addition, differences between CAD studies and clinical trials must be recognised and addressed. Minorities are historically underrepresented in medical research, and this can limit the generalisability of research findings. Encouraging participation in clinical trials can provide valuable information about the efficacy and safety of CAD interventions in different populations. Collaboration between healthcare providers, policymakers, and community organisations is essential to address disparities in CAD management. Implementing targeted interventions that address socioeconomic factors, cultural competence, and research engagement can significantly reduce disparities in CAD management [28].

Long-term outcomes and follow-up

Developments in CAD monitoring have led to enhanced temporary results, such as fewer step-by-step difficulties and medical facility readmissions. Nevertheless, comprehending the treatment's toughness and adherence efficiency is essential to enhancing a person's treatment [29]. Comprehensive research involving significant professional tests and real-world computer registries is needed to assess the performance of brand-new treatments and enhance existing techniques [30]. They provide valuable information about the risk of recurrent events, the need for secondary prevention, and the impact of lifestyle changes on long-term prognosis. Follow-up care is central to managing CAD, allowing healthcare providers to monitor patient progress, address ongoing risk factors, and assess follow-ups. Routine follow-ups promote timely changes in the therapy routine and the execution of safety nets, which can gradually impact personal diagnostic results [31].

Moreover, independent learning of the follow-up procedures equips individuals to handle their problems and employ lasting way-of-life adjustments productively. Telemedicine and digital health solutions have transformed follow-up care by providing remote monitoring and increasing patient engagement [32]. Virtual consultations allow healthcare providers to reach patients with barriers to an in-person visit, facilitating ongoing care and quick resolution of any issues. Despite significant advances in managing CAD, ensuring equitable access to follow-up care remains a challenge, particularly for underserved populations. Hence, addressing disparities in follow-up care is vital to improving long-term outcomes for all patients with CAD [33].

**Table 1:** Findings from various sources in tabulated format along with the year of publication and country of origin CAD: coronary artery disease; CVD: cardiovascular disease; LDL: low-density lipoprotein; CKD: chronic kidney disease; PCSK9: proprotein convertase subtilisin/kexin kind 9; CHD: coronary heart disease; CABG: coronary artery bypass grafting; PCI: percutaneous coronary intervention; NNCAs: non-atherosclerotic coronary arteries; MI: myocardial infarction; CCCS: Collaborative Cardiac Care Service; OPCAB: off-pump coronary artery bypass; ICA: invasive coronary angiography; COD: coronary obstructive disease; EF: ejection fraction

Authors	Year	Country	Findings
Malakar et al. [1]	2019	India	CAD is a major global health concern, with a growing presence in developing countries. Limited genetic understanding hinders prevention. Raising awareness, promoting healthy lifestyles, and early diagnosis are vital for CAD management and eradication, necessitating further research.
Thiriet [2]	2019	France	This article proposes strategies to reduce CVD burden and prevent early adverse events in a research-based health system focusing on risk factor reduction and treatment.
Libby and Theroux [3]	2005	Canada	Cardiology's understanding of CAD has evolved significantly. This has practical implications for improved patient care, individualised therapy, and exploring new approaches beyond LDL cholesterol. Preventive interventions and translational research offer opportunities to enhance CAD treatment and outcomes.
Ralapanawa and Sivakanesan [4]	2021	Sri Lanka	The fluctuation in CAD rates can be attributed to factors such as the global adoption of the Western diet, a rise in sedentary lifestyles, advancements in CAD treatment, and enhancements in primary and secondary preventive strategies.
de Oliveira Laterza Ribeiro et al. [5]	2023	Brazil	Advancements in therapeutic and diagnostic strategies have enhanced CAD understanding and treatment. Future research should emphasise stem cell therapies, angiogenic treatments, and improved diagnostics.
Polimeni [6]	2020	Italy	Diagnostic invasive or non-invasive tools and techniques for CAD, management pharmacological or non-pharmacological therapies (like new transcatheter technologies), and treatment of complex clinical scenarios were critically discussed and reviewed in the article.
Kandaswamy and Zuo [7]	2018	USA	This aims to update the literature on these strategies, explaining their diagnostic rationale and CAD treatment potential. Researchers and clinicians worldwide are diligently working on these promising alternatives to existing treatments.
Agrawal et al. [8]	2015	USA	Pharmacological and non-pharmacological management strategies in patients with advanced CKD which are at high risk for the development of CAD and other CVDs.
Tang et al. [9]	2017	China	PCSK9 medication is an innovative treatment that has the potential to directly mitigate the factors contributing to CAD by reducing vascular inflammation and inhibiting the TLR4/NF-κB signalling pathway. PCSK9 may serve as an inflammatory mediator in the development of atherosclerosis, a primary underlying cause of CAD.
Bhatt and Stone [10]	2006	USA	A multifaceted approach is key to preventing and managing angina, using both medication and lifestyle changes to reduce its impact on daily life and exercise. Angina management is best addressed by pharmacologic and lifestyle interventions.
Van Praet et al. [11]	2021	Germany	This article evaluates literature, covering standard approaches and addressing topics like restricted access procedures, patient selection, diagnostics, surgical techniques, anastomotic devices, hybrid coronary revascularisation, and outcome analysis.
Maron et al. [12]	2020	UK	In patients with stable coronary disease and significant ischemia, an initial invasive approach did not lower cardiovascular events or all-cause mortality compared to a conservative approach, with outcomes varying based on the MI definition used.
Kentenich et al. [13]	2023	Germany	Due to assessment inconsistencies, we must interpret guideline adherence results cautiously. This review examines healthcare providers' adherence to chronic CAD and myocardial revascularisation guidelines in the literature.
Gaudel et al. [14]	2022	Finland	The counselling intervention effectively addressed multiple lifestyle-related risk factors, including dietary habits, physical activity, stress, overweight/obesity, smoking, alcohol use, and medication adherence. This highlights the importance of further research into these lifestyle risk factors and effective interventions.
Rutledge et al. [15]	1999	USA	Multidisciplinary lifestyle modification programmes that target cardiovascular risk factors are recognised for their substantial influence on cardiac risk factors in individuals with coronary atherosclerosis. The adherence of participants to these self-regulated programmes is a crucial consideration.
Giuliano et al. [16]	2017	Australia	Enhancing management continuity ensures uniform, evidence-based care for CAD patients, while improving relational continuity boosts patient engagement with a dedicated healthcare team for long-term cardiac health.
Short et al. [17]	2021	USA	CVD causes global health problems, but lifestyle changes and medication offer opportunities to help millions. Despite medical progress, two-thirds of patients do not follow treatment, even in secondary prevention.
Jankowski et al. [18]	2018	USA	Cardiovascular risk control in CAD patients has seen limited improvement, with the greatest potential for better outcomes observed in patients under primary care. This improvement is tied to promoting a no-smoking policy and increasing guideline-recommended drug prescriptions.
Sigamani and Gupta [19]	2022	India	Effective secondary prevention in CHD through adherence to lifestyle changes and medications significantly reduces recurrent coronary events and associated risks.
Narasimhan et al. [20]	2021	USA	In the absence of clear success in human trials, it is vital to standardize study variables, including angiogenic factors, delivery methods, and clinical endpoints. Given the significant potential in treating CVDs, the pursuit of translating animal study efficacy to clinical practice is imperative.
Rao et al. [21]	2015	India	Long-term studies to guide the chronic management of CAD are currently lacking. Consequently, to address the growing CAD epidemic in India, there is an urgent need for comprehensive, high-quality, and enduring research projects that tackle various aspects of the disease. These research endeavours should ultimately pave the way for initiatives aimed at lowering risk factors and enhancing treatment options to alleviate the CAD burden in India.
Damani and Topol [22]	2011	USA	The review explores emerging applications in CAD genomics and pharmacogenomics, offering a glimpse into the groundbreaking discoveries that will unfold in the coming months and years.
Huffman et al. [23]	2017	USA	Collaborative healthcare has proven to be beneficial for individuals with heart disease, as conventional collaborative approaches consistently result in enhancements in mental well-being and overall functioning. There is promise in methods such as coping skills training, mindfulness-based interventions, and positive psychology techniques for patients with heart disease. The development of these programmes will likely involve ongoing improvements in their content and the adoption of innovative delivery methods.
Sandhoff et al. [24]	2008	USA	The service provided by CCCS has garnered satisfaction from both patients and physicians. CCCS has demonstrated ongoing growth and an increased enrolment of patients through the implementation of innovative strategies and technology. These efforts have led to enhanced care and better outcomes for the CAD population.
Mehta et al. [25]	2019	USA	Left main CAD poses the greatest risk in ischemic heart disease. Revascularisation can be done via surgery or percutaneous procedures. A comprehensive heart team discussion is essential when planning multiple interventions. In specific cases where CABG is not feasible, PCI for the left main artery may be an option in complex coronary anatomy.
Mishra et al. [26]	2016	India	A comprehensive approach to managing stable CAD in India, with a focus on risk factor control, lifestyle modifications, and appropriate medical interventions. It emphasises the need for individualised patient care and a multidisciplinary approach to decision-making regarding revascularisation procedures.
Enas et al. [27]	2008	USA	The significantly higher prevalence of CAD in Asian Indians compared to Caucasians, with a focus on the impact on the younger working population. Various factors contributing to this issue are discussed, including lifestyle changes, poor dietary habits, and genetics. The need for aggressive interventions at multiple levels and specific recommendations for CAD prevention among Indians are emphasised.
Sulava and Johnson [28]	2022	USA	Surgery is the standard for treating multivessel or unprotected left main CAD, aiming for complete revascularisation. Minimally invasive, OPCAB, and hybrid methods are considered for patients not suitable for traditional CABG.
Soltani et al. [29]	2021	Iran	The short- and long-term results of patients who undergo CABG at a high-potential hospital are like those at many other centres. Nevertheless, further investigations are required to enhance these outcomes.
Kimura et al. [30]	2002	Japan	The effectiveness and safety of coronary stenting appeared to be maintained in a clinical context for a period of seven to 11 years. Nonetheless, there was a frequent occurrence of late luminal renarrowing beyond the four-year mark, indicating the necessity for extended follow-up.
Hanson et al. [31]	2021	USA	Long-term follow-up of patients undergoing non-emergent coronary angiography found similar low cardiac event rates for those with NNCAs and nonobstructive CAD, but higher rates in obstructive CAD patients. Increased use of medications post ICA may explain the low event incidence.
Tomai et al. [32]	2019	Italy	Surgical, endovascular, and hybrid treatments for CAD and COD offer similar five-year outcomes. Surgical treatment has the lowest mortality, while hybrid treatment shows a slightly higher risk of MI. Overall, results are favourable compared to natural disease progression.
Cole et al. [33]	2003	USA	Coronary disease in young adults has a poor prognosis with factors like prior MI, diabetes, smoking, and low EF leading to higher mortality. Understanding unique atherosclerosis in young patients is essential, emphasising known risk factors, and urgent treatment, along with the need for more research.

## Conclusions

Continued innovations in managing CAD have led to significant advances that promise to improve patient outcomes and reduce the global burden of this common cardiovascular disease. With CAD's profound global impact, researchers, practitioners, and policymakers have collaborated to revolutionise its diagnosis, treatment, and prevention. The diagnostic landscape has been redefined as non-invasive imaging techniques like CCTA and CMR grant clinicians unprecedented insight into coronary anatomy and function. Biomarkers and personalised risk profiling enhance early detection and tailored intervention strategies, ushering in an era of precision medicine. Pharmacological interventions have also seen remarkable strides, boasting potent antiplatelet agents, lipid-lowering therapies, and angina treatments that have rewritten the trajectory of CAD management. These breakthroughs and precision medicine empower practitioners to craft personalised therapeutic regimens that optimise efficacy and minimise adverse effects. Invasive procedures have been revitalised, exemplified by PCI advancements like drug-eluting stents and bioresorbable scaffolds. Intravascular imaging techniques amplify the precision of interventions, while minimally invasive methods reduce procedural risks. Emphasising holistic well-being, lifestyle changes, and cardiac rehabilitation have emerged as formidable allies against CAD. Integrating telemedicine and digital health solutions further augments care, empowering remote monitoring, early detection, and patient engagement. Secondary prevention strategies underscore a nuanced understanding of patient risks, integrating genetic insights and targeted therapies into the management approach. Collaborative care models unite diverse experts, providing comprehensive, patient-centric strategies. As disparities persist, strides have been made to address them, promoting inclusivity and equitable access to cutting-edge care. The commitment to research, evidenced by genetic therapies, targeted interventions, and regenerative medicine, underscores an unwavering dedication to CAD's conquest. In summation, the mosaic of CAD management encapsulates innovation, collaboration, and patient centricity. With each stride forward, the goal of minimising CAD's global impact inches closer, reinvigorating the medical community's resolve to craft a healthier future for all affected by chronic cardiovascular diseases or syndromes.
